# Plant‐produced encapsulin displays non‐typhoidal *Salmonella enterica* antigens and assembles into mosaic nanoparticles

**DOI:** 10.1111/febs.70340

**Published:** 2025-11-20

**Authors:** Carly A. Charron, Angelo Kaldis, Shabnam Shamriz, Justin B. Renaud, Moussa S. Diarra, Christopher P. Garnham, Rima Menassa

**Affiliations:** ^1^ Biology Department Western University London ON Canada; ^2^ London Research and Development Centre Agriculture and Agri‐Food Canada London ON Canada; ^3^ Guelph Research and Development Centre Agriculture and Agri‐Food Canada Guelph ON Canada; ^4^ Biochemistry Department Western University London ON Canada; ^5^ Present address: Guelph Research and Development Centre Agriculture and Agri‐Food Canada Guelph, ON Canada

**Keywords:** encapsulin, plant molecular farming, protein nanoparticle, *Salmonella*, subunit vaccine

## Abstract

Foodborne illnesses have major public health and economic impacts. Non‐typhoidal *Salmonella enterica* serovars (*Salmonella*), associated with the consumption of contaminated poultry products, are among the most common causes of foodborne illness in humans. Vaccination of poultry flocks is one of the main methods to prevent *Salmonella* infection in poultry; however, the vaccines currently available have limitations. Because of this, there is an urgent need for more effective *Salmonella* vaccines. Ferric enterobactin receptor (FepA) is a *Salmonella* outer‐membrane protein that shows promise as a vaccine antigen due to its important role in iron acquisition and its ubiquity on the surface of *Salmonella*. Here, we describe the production of encapsulin nanoparticles displaying antigenic epitopes from FepA in *Nicotiana benthamiana*. Five FepA–encapsulin fusion proteins were transiently expressed in *N. benthamiana* leaves and targeted to either the chloroplast or the cytosol. We found that accumulation was highest in the chloroplast, with levels over 0.7 mg·g^−1^ of leaf fresh weight. Encapsulin nanoparticles self‐assembled within plant cells and FepA epitopes were displayed on their surface, as confirmed by transmission electron microscopy and immunogold labeling. Differentially tagged encapsulin monomers were also co‐infiltrated into the leaves of *N. benthamiana*, where they assembled into mosaic nanoparticles displaying each of the different tags. Encapsulin and one FepA–encapsulin fusion protein were also expressed transplastomically in *Nicotiana tabacum*, where they self‐assembled into nanoparticles and the fusion protein accumulated to 2.4 mg·g^−1^ of leaf fresh weight, nearly fivefold higher than in *N. benthamiana.* This study highlights the capability of encapsulin for use in vaccine design.

## Graphical abstract

This study investigates the potential of encapsulin as an antigen display platform for the development of a candidate *Salmonella* vaccine for poultry. Encapsulin constructs were transiently expressed in *Nicotiana benthamiana* where they accumulated to high levels. All of the encapsulin constructs assembled into nanoparticles displaying antigens on their surface, and differentially tagged encapsulin proteins assembled into mosaic nanoparticles displaying each of the different tags.

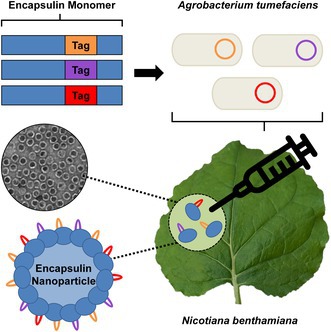

**Abbreviations**
BSA: bovine serum albumin
E: encapsulin
E‐cMyc: encapsulin‐cMyc construct
E‐HA: encapsulin‐HA construct
E‐L10: FepA loop 10–encapsulin fusion construct
E‐L2: FepA loop 2–encapsulin fusion construct
E‐L3: FepA loop 3–encapsulin fusion construct
E‐L4: FepA loop 4–encapsulin fusion construct
E‐L8: FepA loop 8–encapsulin fusion construct
ELISA: enzyme‐linked immunosorbent assay
ER: endoplasmic reticulum
FepA: ferric enterobactin receptor
FW: fresh weight
HA: hemagglutinin
IgA: immunoglobulin A
IgG: immunoglobulin G
IMAC: immobilized metal affinity chromatography
L10: FepA loop 10
L2: FepA loop 2
L3: FepA loop 3
L4: FepA loop 4
L8: FepA loop 8
LC‐MS: liquid chromatography‐mass spectrometry
PBS: phosphate‐buffered saline
pCLGG‐X: pCamLiteGoldenGate‐X
RBC‐S‐TP: chloroplast transit peptide from the small subunit of RuBisCo
SDS/PAGE: sodium dodecyl sulfate/ polyacrylamide gel electrophoresis
SEC: size‐exclusion chromatography
tCUP: tobacco cryptic upstream promoter translational enhancer
UA: uranyl acetate

## Introduction

Foodborne illnesses represent significant public health and economic risks [1, 2]. Non‐typhoidal *Salmonella* are among the leading causes of foodborne illness in humans and are estimated to cause 95 million cases per year worldwide [3]. Poultry is one of the main reservoirs for *Salmonella* and over 50% of all human salmonellosis cases have been associated with the consumption of contaminated poultry products [4]. Antibiotics have historically been used for controlling infection in poultry; however, with the rise of antibiotic‐resistant bacteria including *Salmonella*, new control measures are needed [5, 6]. Vaccination of poultry flocks is now considered the most effective *Salmonella* control measure; however, the vaccines currently available have limitations such as safety concerns, poor immunogenicity, and inefficient methods of administration [7, 8]. Thus, there is a need for practical and efficient vaccines.

The outer membrane of *Salmonella* harbors many proteins that have shown potential as antigens for vaccine development [7, 9, 10]. Here, we have chosen to utilize the ferric enterobactin receptor (FepA), a *Salmonella* outer membrane protein involved in iron acquisition, a process that is essential for *Salmonella* survival and virulence within the host [11, 12]. *Salmonella* acquire and solubilize iron using small iron‐chelating molecules called siderophores and the iron‐siderophore complexes are imported into the cell by iron acquisition proteins including FepA [11, 13, 14]. FepA has a beta‐barrel structure that spans the outer membrane and contains large extracellular loops that interact with the iron‐siderophore complexes [15, 16]. The extracellular loops of FepA are attractive target antigens as they are surface exposed on the bacteria where they would be visible to the immune system, and antibodies that recognize and bind to them may inhibit bacterial iron uptake and prevent *Salmonella* from proliferating within the host [17].

Protein nanoparticles have recently emerged as effective display platforms for vaccine antigens [18, 19]. Protein nanoparticles are multisubunit proteins that self‐assemble into stable, complex structures, and can display antigenic epitopes on their exterior surface in a highly organized and repetitive manner, similar to the native pathogen [20]. Encapsulin is a protein nanoparticle produced by the thermophilic bacterium *Thermotoga maritima* and it is assembled from 60 copies of identical 31 kilodalton (kDa) monomers [21]. The encapsulin monomer was reported to contain six sites, which are amenable to modification, leading to the production of stable and/or soluble encapsulin nanoparticles [22]. Of these sites, Site 138–139 has been the most extensively studied and genetic modifications at this position have been previously co‐opted for vaccine design and drug delivery [23, 24, 25].

Encapsulin has been recombinantly produced in insect cells [26] and *Escherichia coli* [25, 27, 28, 29], but here we report the first recombinant production of encapsulin in plants. Plants are easy and inexpensive to grow and maintain, and expressing recombinant proteins transiently in plants allows for fast and easily scalable production. Plant production also provides the potential for oral delivery of veterinary vaccines through the addition to animal feed. This could eliminate risks associated with parenteral administration and allow for efficient and cost‐effective mass vaccination. Oral delivery could also reduce vaccine production costs by eliminating expensive and time‐consuming protein purification steps [30, 31].

In this paper, we identify a novel insertion site in encapsulin and we describe the plant production and characterization of encapsulin and five FepA‐encapsulin fusion proteins displaying different FepA epitopes. We found that both encapsulin and the FepA‐encapsulin fusions accumulate and assemble in the chloroplast and cytosol of *Nicotiana benthamiana*, as well as the chloroplast of *Nicotiana tabacum*. Additionally, we show that the FepA epitope loop 2 is surface‐displayed on assembled nanoparticles. Furthermore, we show for the first time that differentially tagged encapsulin monomers co‐assemble in *N. benthamiana*, forming mosaic nanoparticles displaying each of the different tags. These results highlight the potential of encapsulin as a display platform for the development of a candidate *Salmonella* vaccine.

## Results

### Encapsulin accumulates to high levels and assembles into nanoparticles when expressed in plants

**Fig. 1:**
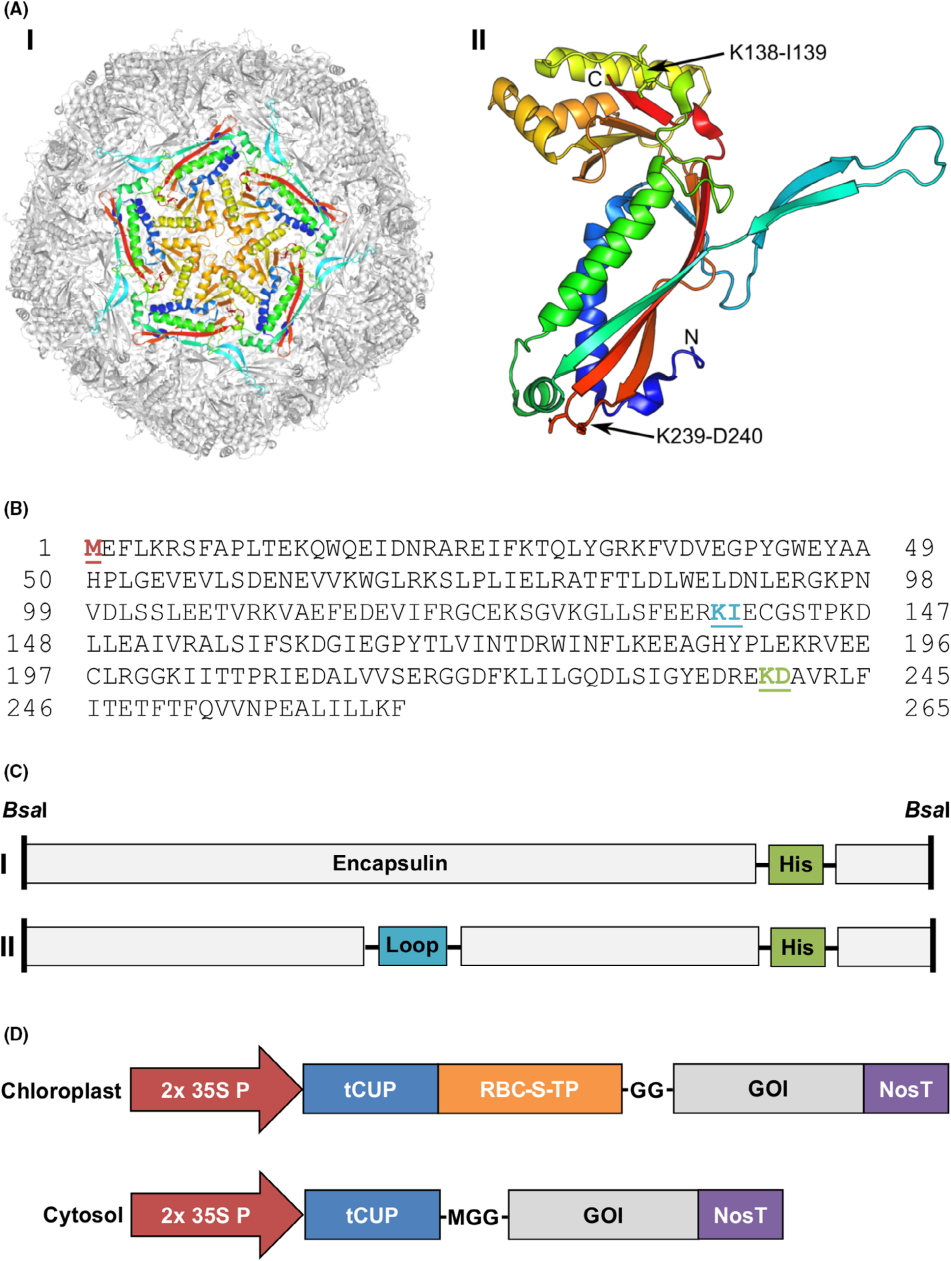
Construct design and pCLGG‐X plant protein expression vectors. (A) Assembled encapsulin 60‐mer with a pentamer highlighted (I) and encapsulin monomer with the C terminus (C), N terminus (N), and insertion points (K138‐I139 and K239‐D240) indicated (II). Structures generated using PyMOL (The PyMOL Molecular Graphics System) (B) Reference amino acid sequence for the encapsulin monomer from Protein Data Bank (PDB: 3DKT). The N‐terminal methionine (red) was removed prior to gene synthesis, as a start codon is encoded in the pCamLiteGoldenGate‐X (pCLGG‐X) vectors. The insertion point (between residues K138‐I139) of the Ferric enterobactin receptor (FepA) loops is indicated in blue, and the insertion point (between residues K239‐D240) of the His‐tag is indicated in green. (C) Encapsulin alone (I) and FepA‐encapsulin fusion construct (II). Gray boxes represent the encapsulin amino acid sequence. The FepA loops (blue) and the His‐tag (green) are flanked by the linker sequence (G_4_S) to allow for flexibility (horizontal black lines). The gene is flanked by *Bsa*I restriction enzyme sites (vertical black lines) for Golden Gate cloning. (D) pCLGG‐X plant expression vectors target proteins to the chloroplast or cytosol. Both vectors contain the double‐enhanced *35S* promoter (red), *tCUP* translation enhancer (blue), and *Nos* terminator (purple). The transit peptide from the small subunit of RuBisCo (RBC‐S‐TP; orange) is included in the chloroplast vector to target the proteins to the chloroplast stroma. Cloning into the chloroplast vector results in the addition of two N‐terminal glycines to the mature encapsulin proteins, while cloning into the cytosol vector results in the addition of a methionine and two glycines to the N terminus. GOI, gene of interest.

*Thermotoga maritima* encapsulin is a multimeric protein that assembles into large icosahedral nanocages approximately 25 nm in diameter. The encapsulin monomer first assembles into a pentamer, and 12 pentamers assemble to form the 60‐mer structure. Encapsulin monomers contain three distinct domains (P, A, and E). Visual inspection of the protein within its fully assembled 60‐mer structure (PDB: 3DKT) identified several previously reported surface‐exposed loops with potential for genetic insertion of affinity tags and antigenic epitopes. Further analysis of the structure identified two additional surface‐exposed regions on the protein. These include position K239‐D240 that resides in a loop that links strands β10 and β11 within the P‐domain, and position K138‐I139 that constitutes a small 2‐residue β strand (β5) that caps the top end of the 5‐stranded β‐sheet within the A domain (Fig. 1A). Position K239‐D240 had never previously been tested for genetic insertion of affinity tags and/or antigens.

To assess whether encapsulin could be produced in plants, the *T. maritima* encapsulin sequence (PDB: 3DKT) was modified by inserting a 6xHis‐tag flanked by flexible linkers (G_4_S) at position K239‐D240 to allow for protein detection and purification (Fig. 1B; Fig. 1CI). While detection tags are often included at the N‐ or C‐terminus of a protein [32], the N‐terminus of encapsulin projects to the interior of the assembled structure [21], and thus is not accessible, while the C‐terminus has been reported to have limited accessibility as well [24]. Therefore, we chose position K239‐D240 as this position is surface‐exposed on assembled encapsulin, allowing the protein to be detected in either its native or denatured state. This modified sequence was then codon optimized for nuclear expression in *N. benthamiana* and cloned into plant expression vectors (Fig. 1D), which add two N‐terminal glycine residues to stabilize the protein according to the N‐end rule [33].

**Fig. 2:**
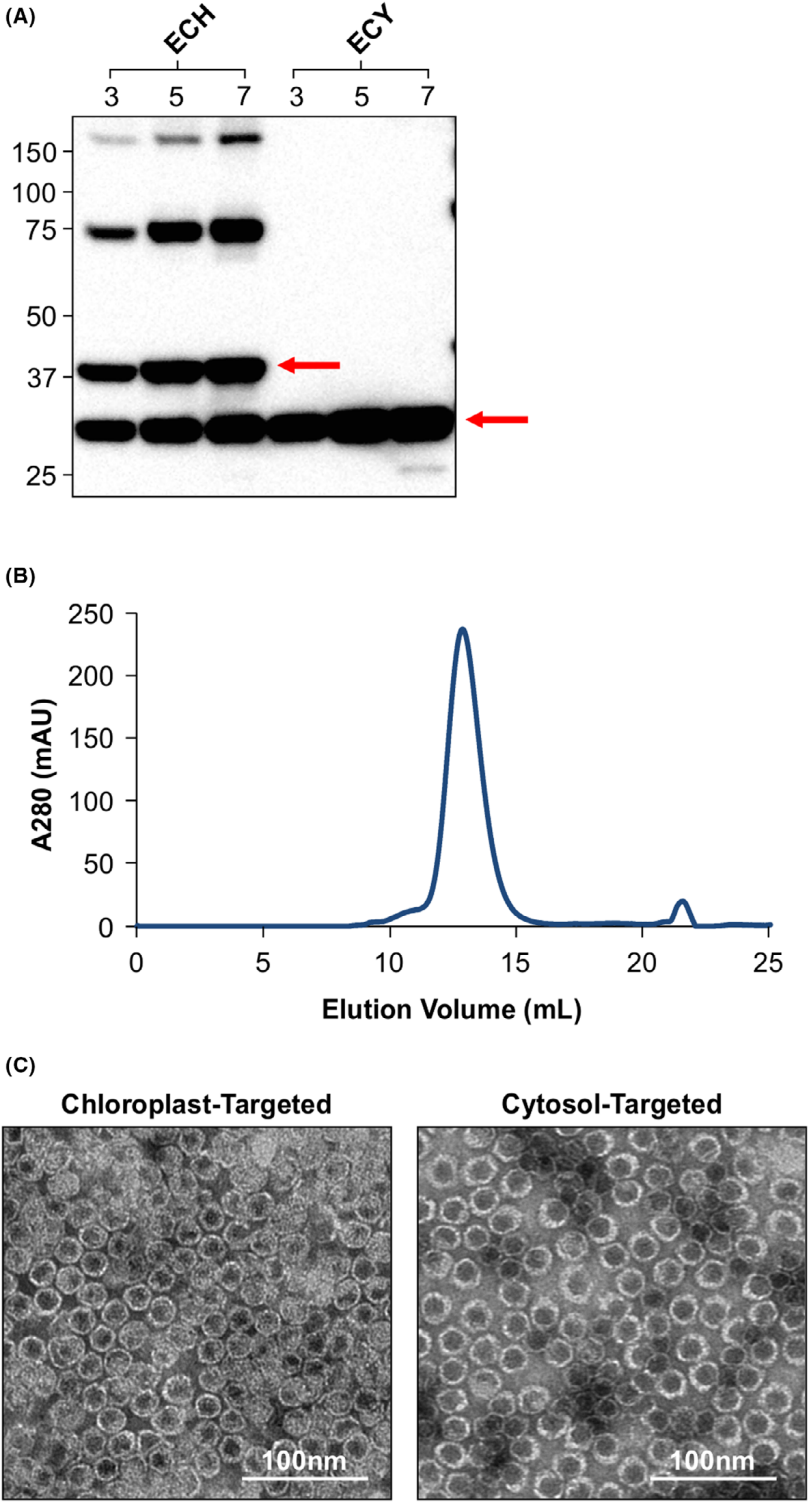
Plant‐produced encapsulin accumulates and assembles in the chloroplast and cytosol. All analyses were repeated twice. (A) Proteins were targeted to the chloroplast (ECH) and the cytosol (ECY) and leaf samples were collected on Days 3, 5, and 7 postinfiltration. All lanes were loaded with total soluble protein extracted from 1.6 mg of leaf tissue. Proteins were detected using an anti‐His primary antibody (1:5000). The recombinant protein is indicated by red arrows. Numbers on the left‐hand side of the blot represent the size (kDa) of the molecular weight markers. The expected molecular weight of the encapsulin (E) monomer is 31.7 kDa. (B) SEC analysis of IMAC‐purified cytosol‐targeted encapsulin. One major peak was observed on the chromatogram corresponding to the 1.902‐MDa encapsulin nanoparticle. (C) Negative‐stained transmission electron micrographs of chloroplast‐targeted and cytosol‐targeted encapsulin obtained using a JEOL JEM‐1400Flash (JEOL Ltd.) microscope operated at 80 kV. Images were taken at 50000× magnification. Size bar represents 100 nm. IMAC, immobilized metal affinity chromatography; SEC, size‐exclusion chromatography.

To evaluate encapsulin expression, the recombinant protein was transiently expressed in *N. benthamiana* using agroinfiltration. Prokaryotes, such as *T. maritima*, have limited protein processing complexes and capacities to perform posttranslational modifications [34], whereas plants have the capacity to perform a variety of posttranslational modifications, especially when proteins are targeted to organelles along the secretory pathway, such as the ER, where there are numerous chaperones that can aid in modifications such as glycosylation [35, 36]. Because encapsulin is a prokaryotic protein, we targeted the recombinant protein to the cytosol and chloroplast, as both subcellular compartments lack the machinery for complex posttranslational modifications [37, 38], and thus, the recombinant protein should be processed in a prokaryotic‐like manner. The rate of protein synthesis and degradation varies on a gene‐by‐gene basis [39]; therefore, a one‐week time‐course was conducted to assess the accumulation level of encapsulin over time to determine its accumulation profile. Samples were collected on Days 3, 5, and 7 post‐infiltration, as gene products typically reach their peak level during this period [36]. There was a slight increase in encapsulin accumulation over time, with more protein accumulating on Day 7, indicating that encapsulin is a stable protein (Fig. 2A).

A double‐banding pattern, at ~31 kDa and ~37 kDa, which could correspond to the mature protein and the immature protein with an intact transit peptide, as well as a ladder of larger bands were observed for chloroplast‐targeted encapsulin. The larger bands likely correspond to assembled intermediates such as dimers (~75 kDa) and tetramers (~150 kDa). A single band at ~31 kDa, corresponding to the expected molecular weight of encapsulin, was observed when the recombinant protein was targeted to the cytosol.

To assess quaternary assembly and nanoparticle formation, encapsulin was extracted and purified from leaf tissue infiltrated with encapsulin targeted to the cytosol or to the chloroplast using immobilized metal affinity chromatography (IMAC) followed by size‐exclusion chromatography (SEC) (Fig. 2B). One major peak was observed on the SEC chromatogram, corresponding to the 1.902‐MDa encapsulin nanoparticle, indicating that the majority of the protein is assembling into nanoparticles. The purified samples were then visualized using transmission electron microscopy. Uniformly shaped, round nanoparticles, approximately 22 nm in diameter, were observed in both samples (Fig. 2C). This is the first time that encapsulin assembly has been demonstrated in plants.

### Fusion of *Salmonella* epitopes does not impede encapsulin accumulation or nanoparticle assembly

Five extracellular loops from the iron‐acquisition protein FepA were selected as vaccine epitopes as they are surface‐exposed on *Salmonella* and play an important role in iron‐acquisition, which is essential for *Salmonella* survival and virulence. The crystal structure of *Salmonella* FepA (GenBank accession no. AM933172.1) has not been solved; therefore, structure prediction was performed using Phyre2 and sequence alignment with the homologous FepA protein from *E. coli*. Eleven extracellular loops were identified and Loops 2, 3, 4, 8, and 10 (Table 1) were selected as the best candidate epitopes based on their size, predicted antigenicity, sequence diversity from commensal gut bacteria, and sequence conservation across *Salmonella enterica* serovars. The loop coding sequences were individually inserted at position K138‐I139 of the modified encapsulin monomer, with flexible linkers flanking both sides of the sequence, and a 6xHis‐tag flanked by flexible linkers was inserted at position K239‐D240 for protein detection and purification (Fig. 1B, Fig. S1CII). A total of five FepA‐encapsulin fusion constructs (E‐L2, E‐L3, E‐L4, E‐L8, and E‐L10) were synthesized and cloned into chloroplast‐ and cytosol‐targeting plant expression vectors (Fig. 1D).

**Table 1:** Extracellular loops identified and utilized from FepA.

FepA loop	Amino acid sequence	Amino acid length
Loop 2 (L2)	QADARNINQGHQSERTGSYADTLPAGRE	28
Loop 3 (L3)	YAGDTQNTNTNQLVKDNYGKET	22
Loop 4 (L4)	MPEGLAGGTEGIFDPKASQKYAD	23
Loop 8 (L8)	GTVPLQRINNGKTDVYQ	17
Loop 10 (L10)	GKQEPKKYDYQGNPVTGTDKQAVSP	25

**Fig. 3:**
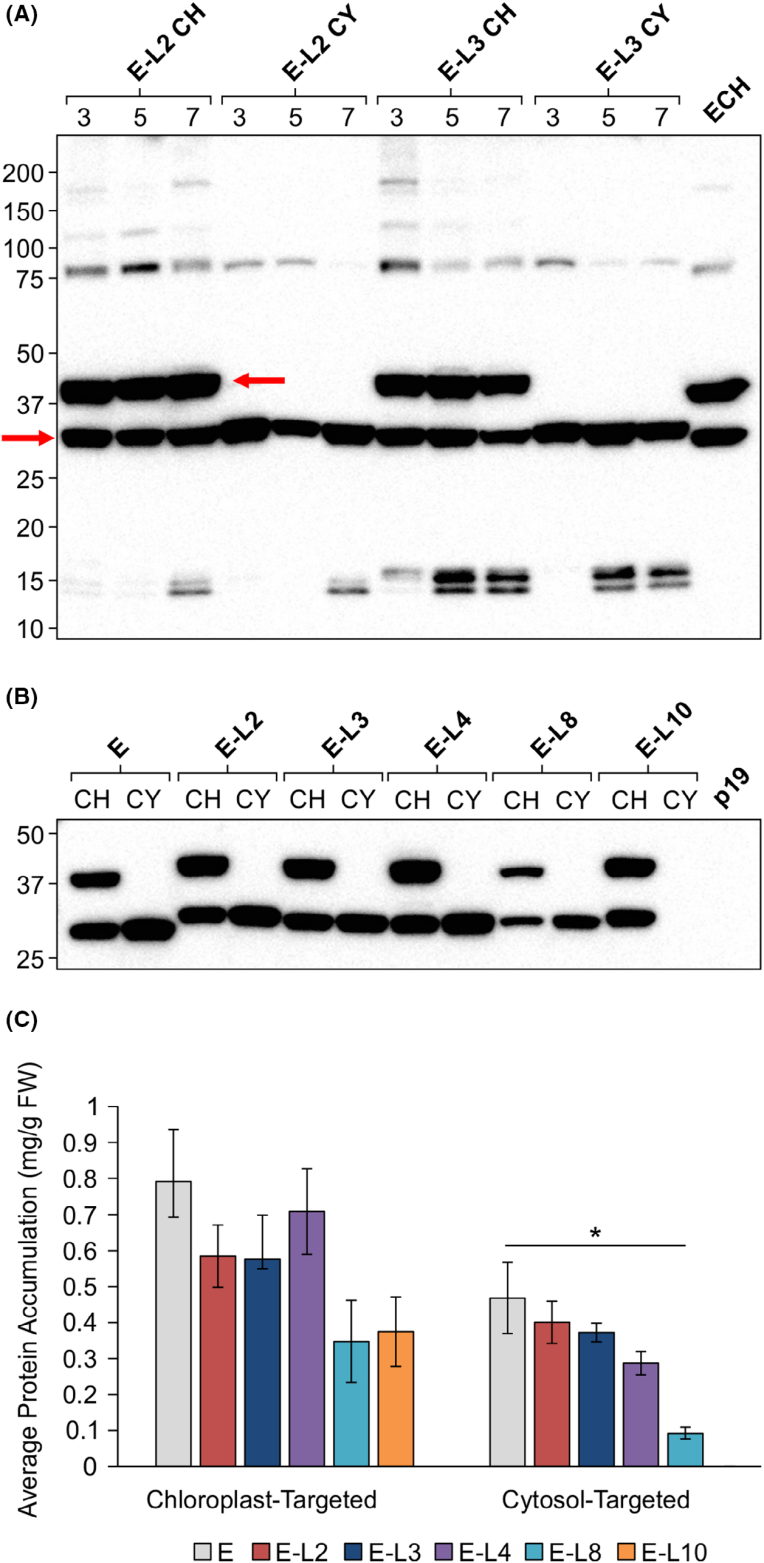
Plant‐produced FepA‐encapsulin fusion proteins accumulate in the chloroplast and cytosol. (A) Proteins were targeted to the chloroplast (CH) and the cytosol (CY) and leaf samples were collected on Days 3, 5, and 7 postinfiltration. All lanes were loaded with total soluble protein extracted from 2.0 mg of leaf tissue. Proteins were detected using an anti‐His primary antibody (1:5000). The recombinant protein is indicated by red arrows. Numbers on the left‐hand side of the blot represent the size (kDa) of the molecular weight markers. The expected molecular weight (kDa) of E‐L2 is 35.5, E‐L3 is 34.9, and E is 31.7. Results are representative of two replicates. (B) Proteins were targeted to the chloroplast (CH) and cytosol (CY) and were sampled on Day 3 postinfiltration. All lanes were loaded with total soluble protein extracted from 1.6 mg of leaf tissue. As a negative control, total soluble protein extract was loaded from plants infiltrated with *p19*, a suppressor of post‐transcriptional gene silencing. Numbers on the left‐hand side of the blot represent the size (kDa) of the molecular weight marker. The expected molecular weights (kDa) of the monomers are as follows: E: 31.7, E‐L2: 35.5, E‐L3: 34.9, E‐L4: 34.3, E‐L8: 33.8, and E‐L10: 35.2. Proteins were detected using an anti‐His primary antibody (1:5000). (C) Average recombinant protein accumulation levels for chloroplast‐targeted proteins and cytosol‐targeted proteins in mg·g^−1^ leaf fresh weight (mg·g^−1^ FW). All samples were collected on Day 3 post‐infiltration. *N* = 14. Accumulation levels were averaged across biological replicates (± standard error of the mean) and significance (*P‐value <0.05) was determined by ANOVA followed by a Tukey's *post hoc* test. Cytosol‐targeted E‐L10 was excluded as no accumulation was observed for this recombinant protein. E, encapsulin; FepA, Ferric enterobactin receptor; E‐L#, Respective FepA loop–encapsulin fusion protein.

A one‐week time course was also conducted to assess the accumulation profile of the FepA‐encapsulin fusion proteins targeted to the cytosol and the chloroplast. The level of recombinant protein remained relatively consistent over time; however, the appearance of degradation products (~15 kDa) increased as time progressed (Fig. 3A). Because of this, Day 3 post‐infiltration was determined to be the optimal time to collect leaf samples for all the FepA‐encapsulin fusion proteins.

To assess whether the insertion of FepA epitopes impacts the accumulation of encapsulin, all the constructs were transiently expressed in *N. benthamiana* (Fig. 3B). Recombinant protein accumulation was observed for all the FepA‐encapsulin fusion proteins except for E‐L10 targeted to the cytosol. Despite multiple attempts at expression, no accumulation was observed. The construct was sequenced, and no errors were identified; therefore, this protein likely accumulates to undetectably low levels in the cytosol.

As seen for encapsulin, a double‐banding pattern was also observed for all FepA‐encapsulin fusion proteins targeted to the chloroplast, while a single band was observed when the recombinant proteins were targeted to the cytosol. A ladder of larger bands was also visible for both the chloroplast‐ and cytosol‐targeted proteins, which likely correspond to assembled intermediates. To investigate the nature of the upper band in the chloroplast‐targeted proteins (~37 kDa), LC–MS intact mass analysis was performed on purified cytosol‐ and chloroplast‐targeted E‐L2. The theoretical mass of E‐L2 is 35569.91 Da [40]. Two highly similar proteins were identified in the cytosol‐targeted E‐L2 sample with masses of 35569.0 ± 1.1 Da and 35583.3 ± 1 Da. The higher molecular weight protein (+ 14.3 ± 1.5 Da) could be due to potential post‐translational modifications such as methylation, which would add approximately 14 Da to the protein [40, 41]. Two proteins were identified in the chloroplast‐targeted E‐L2 sample with masses of 35582.3 ± 0.6 Da and 41244.7 ± 1.7 Da. The theoretical mass of the chloroplast transit peptide (transit peptide from the small subunit of RuBisCo) is 5769.6 Da [40]; therefore, we could infer that the two proteins detected in the chloroplast‐targeted E‐L2 sample correspond to the unprocessed protein containing the entire transit peptide and the mature processed protein.

The accumulation of encapsulin and the FepA‐encapsulin fusion proteins was assessed by quantitative immunoblotting of 15 biological replicates, where the intensity of both chloroplast bands or the single cytosol band were quantified. All the recombinant proteins accumulated to high levels with the chloroplast‐targeted proteins ranging from 0.35–0.79 mg·g^−1^ fresh weight (FW), and the cytosol‐targeted proteins ranging from 0.09–0.47 mg·g^−1^ FW (Fig. 3C). In the case of the chloroplast‐targeted proteins, epitope insertion had no significant impact on encapsulin accumulation (*P*‐value <0.05) while of the cytosol‐targeted proteins, only E‐L8 accumulated significantly less than encapsulin alone (*P*‐value <0.001).

**Fig. 4:**
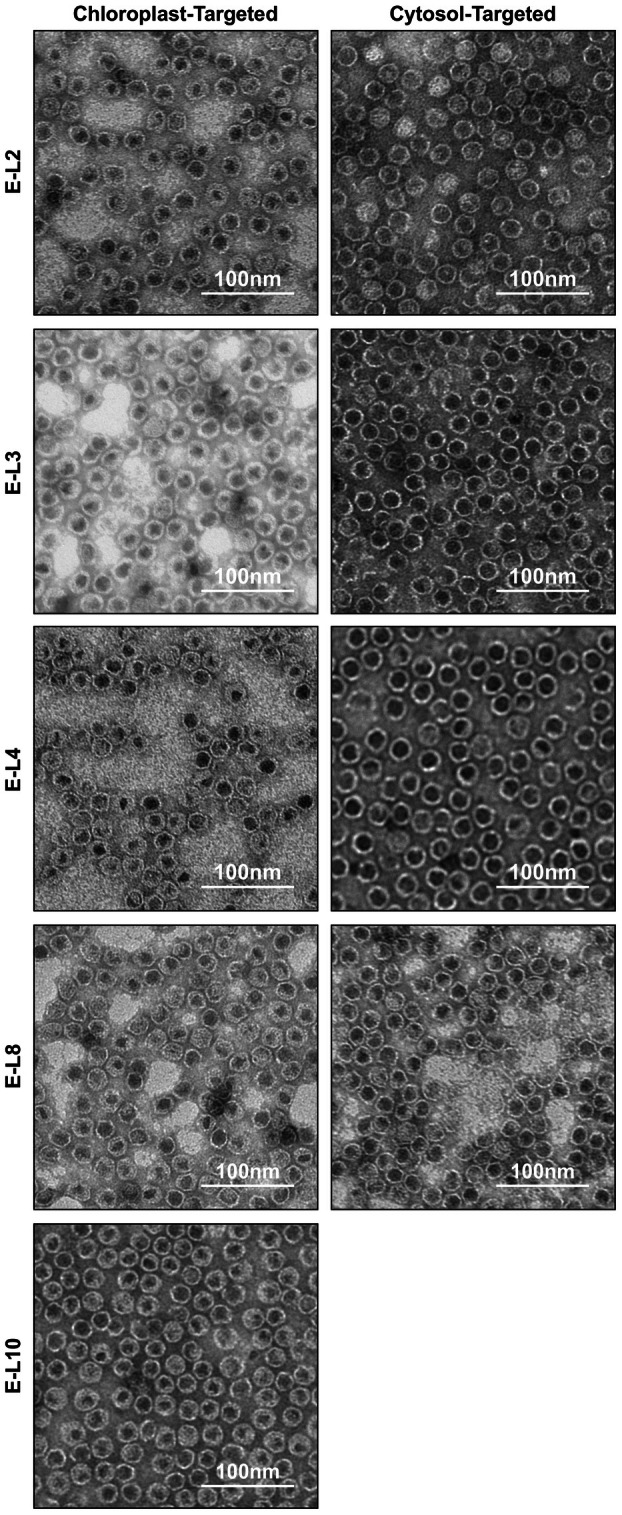
Plant‐produced FepA‐encapsulin fusion proteins assemble into nanoparticles. Negative‐stained transmission electron micrographs of chloroplast‐targeted and cytosol‐targeted FepA‐encapsulin fusion proteins were obtained using a JEOL JEM‐1400Flash (JEOL Ltd.) microscope operated at 80 kV. Images were taken at 50 000× magnification. Size bars represent 100 nm. Images are representative of the entire grid and similar results were visualized across two replicates. FepA, Ferric enterobactin receptor; E, Encapsulin; E‐L#, Respective FepA loop–encapsulin fusion protein.

**Fig. 5:**
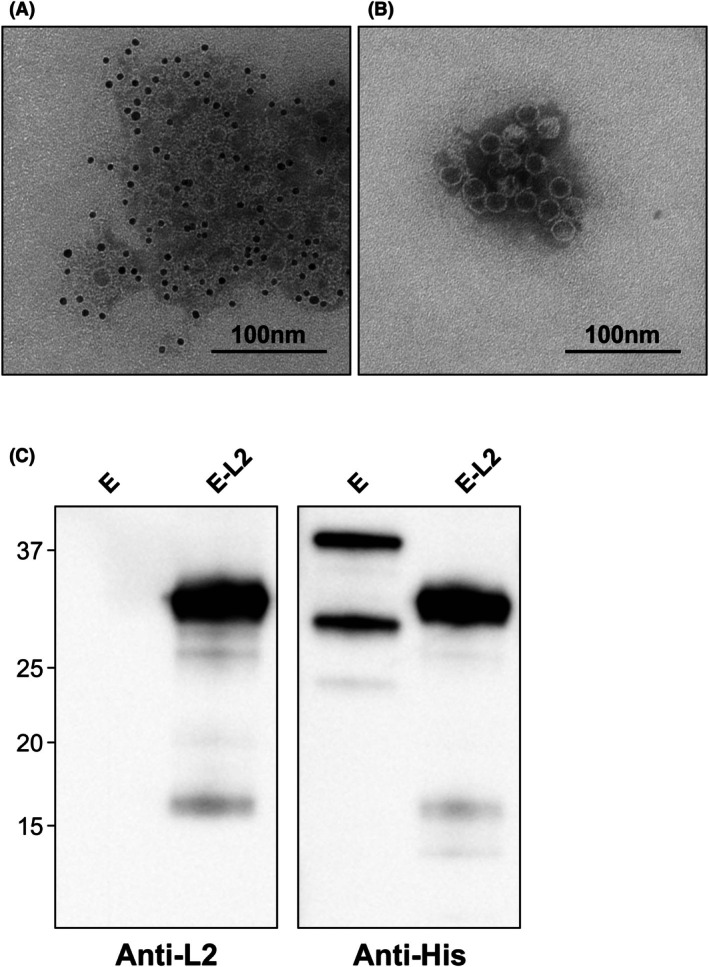
FepA epitopes are surface displayed on assembled encapsulin nanoparticles. Purified chloroplast‐targeted E‐L2 was immunolabeled, negatively stained, and examined using a JEOL JEM‐1400Flash (JEOL Ltd.) microscope operated at 80 kV. Both images were taken at 60 000× magnification. Size bars represent 100 nm. (A) Immunogold localization of E‐L2 using an anti‐L2 primary antibody. Gold particles (6 nm) surround nanoparticles indicating that the L2 epitope is displayed on the nanoparticle surface. (B) Negative control for immunogold localization of E‐L2 using no primary antibody for detection. Nanoparticles are present but no gold particles are visible. Images are representative of the entire grid and similar results were visualized across two replicates. (C) Chloroplast‐targeted encapsulin from *N. benthamiana* and E‐L2 from transplastomic *N. tabacum*. Results are representative of two replicates. All lanes were loaded with 50 ng of purified recombinant protein. Proteins were detected using an anti‐L2 (1:5000) or anti‐His (1:5000) primary antibody. Numbers on the left‐hand side of the blot represent the size (kDa) of the molecular weight markers. The expected molecular weight (kDa) of E is 31.7 and E‐L2 is 35.5. FepA, Ferric enterobactin receptor; E, Encapsulin; E‐L2, FepA loop 2–encapsulin fusion protein.

All the FepA‐encapsulin fusion proteins were purified using IMAC and SEC and the purified proteins were analyzed using transmission electron microscopy to assess whether epitope insertion impedes encapsulin assembly. Nanoparticles were observed for all the fusion proteins (Fig. 4), indicating that epitope insertion does not impact encapsulin assembly. Once proper assembly was confirmed, immunogold labeling was performed to assess whether the epitopes are displayed on the exterior surface of encapsulin. For this, a custom primary antibody against FepA loop 2 (L2) was produced in rabbits and incubated with purified E‐L2 nanoparticles, followed by a 6‐nm gold‐labeled anti‐rabbit secondary antibody. Gold particles were observed near E‐L2 fusion proteins (Fig. 5A), indicating that the L2 epitope is displayed on the exterior surface, while no gold particles were visible in the negative control where the primary antibody was omitted (Fig. 5B). To confirm that there is no non‐specific binding of the anti‐L2 antibody to encapsulin, both encapsulin and E‐L2 were immunoblotted with anti‐L2 and anti‐His antibodies. Bands for both proteins were observed when probed with anti‐His, while no band was detectable for encapsulin when probed with anti‐L2 (Fig. 5C). Two bands were present for chloroplast‐targeted encapsulin probed with anti‐His due to retention of the chloroplast transit peptide. Faint, low‐molecular‐weight bands were also present, which likely correspond to degradation products.

### Co‐expression and mosaic‐assembly of encapsulin monomers

**Fig. 6:**
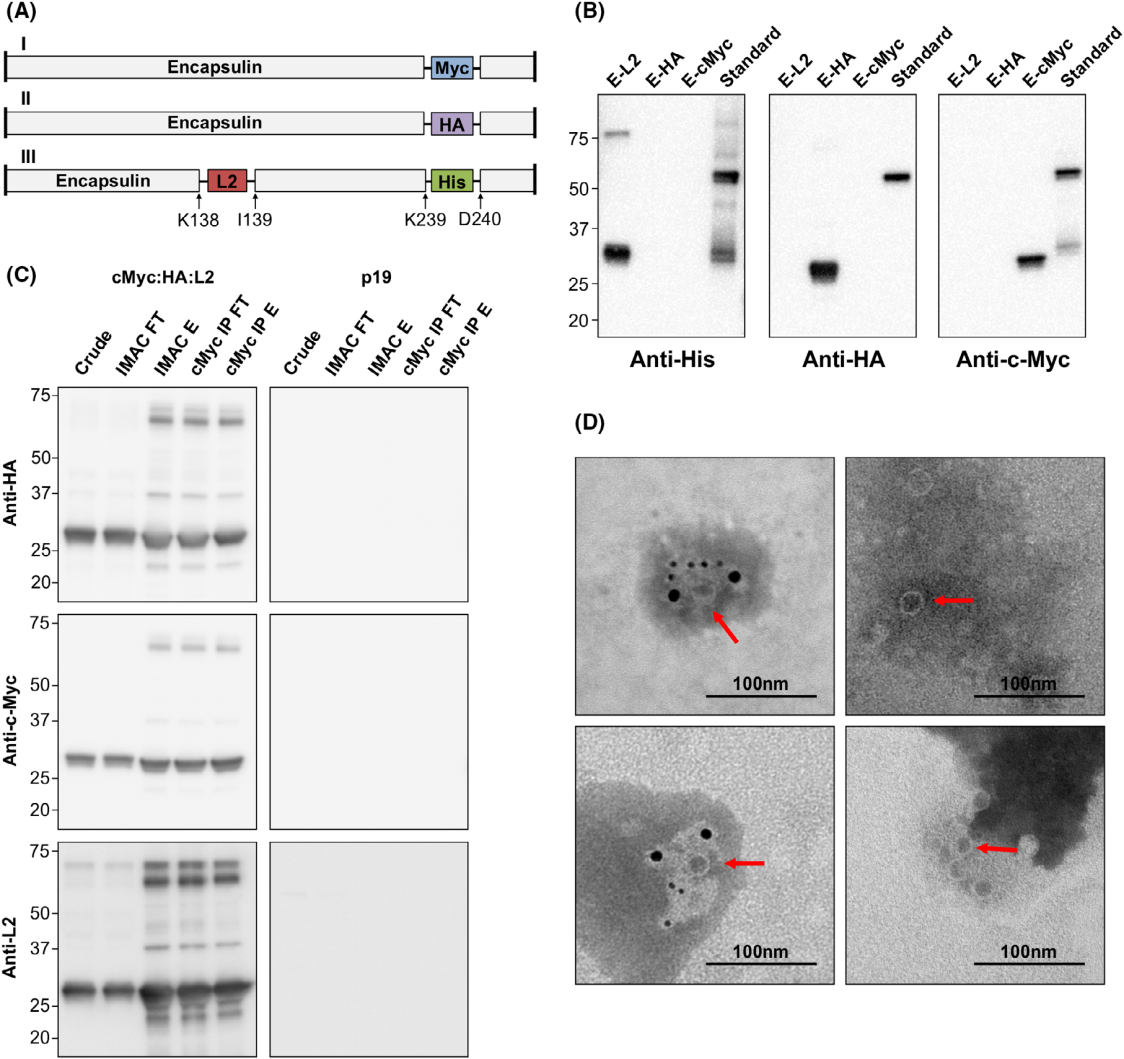
Differentially tagged encapsulin monomers assemble into mosaic nanoparticles. (A) Encapsulin‐cMyc (E‐cMyc) (I) and encapsulin‐HA (E‐HA) (II) were designed and co‐infiltrated with (III) encapsulin‐L2‐His (E‐L2). The numbers along the bottom represent amino acid positions within the encapsulin protein. Myc, c‐myc tag; HA, Hemagglutinin tag. (B) Cytosol‐targeted E‐cMyc, E‐HA, and E‐L2 were transiently expressed in *N. benthamiana*. Total soluble protein extracts from 0.13 mg of leaf tissue were run in triplicate alongside 25 ng of an in‐house protein standard containing a His‐tag, an HA‐tag, and a c‐Myc‐tag. Following transfer to a polyvinylidene fluoride membrane, the blot was cut into three sections and probed separately with anti‐His, anti‐HA, or anti‐c‐Myc primary antibodies. All antibodies were diluted 1:5000. Numbers on the left‐hand side of the blot represent the size (kDa) of the molecular weight markers. The expected molecular weight (kDa) of E‐HA and E‐cMyc is 31.7, and E‐L2 is 35.5 (C) Cytosol‐targeted E‐cMyc, E‐HA, and E‐L2 (cMyc:HA:L2) were co‐expressed in the leaves of *N. benthamiana* plants and samples were collected on Day 3 post‐infiltration. The recombinant proteins were purified via IMAC followed by c‐Myc immunoprecipitation (cMyc IP), and immunoblotted with an anti‐c‐Myc, anti‐HA, or anti‐L2 primary antibody separately. All antibodies were diluted 1:5000. Numbers on the left‐hand side of the blot represent the size (kDa) of the molecular weight markers. Crude, crude extract; IMAC FT, IMAC flow‐through; IMAC E, IMAC elution; cMyc IP FT, cMyc IP flow‐through; cMyc IP E, cMyc IP elution. The same procedure was performed with *N. benthamiana* leaves infiltrated with p19 as a negative control. Results are representative of two replicates. (D) Left panel – purified E‐cMyc, E‐HA, and E‐L2 mosaic nanoparticles were immunolabeled using rabbit anti‐L2 and mouse anti‐c‐Myc primary antibodies (top), or rabbit anti‐L2 and mouse anti‐HA primary antibodies (bottom), and detected with anti‐rabbit antibodies conjugated to 6 nm gold particles, and anti‐mouse antibodies conjugated with 10‐nm gold particles. Right panel ‐ negative controls where primary antibodies were omitted. The proteins were negatively stained and examined using a JEOL JEM‐1400Flash (JEOL Ltd.) microscope operated at 80 kV. Images were taken at 40 000× magnification. Size‐bars represent 100 nm. Red arrows indicate assembled nanoparticles. Images are representative of the entire grid and similar results were visualized across two replicates. FepA, Ferric enterobactin receptor; E‐L2, FepA loop 2–encapsulin fusion protein; L2, FepA loop 2; IMAC, Immobilized metal affinity chromatography.

The rationale for designing FepA‐encapsulin fusion proteins displaying different FepA epitopes was to produce a multivalent vaccine comprised of a mix of the different fusion proteins, to enhance immune targeting of FepA and inhibit *Salmonella* iron uptake and growth. However, expressing and purifying all the proteins is costly, time‐consuming, and labor‐intensive [42]. For these reasons, we sought to determine whether encapsulin monomers containing different epitopes would co‐assemble into mosaic nanoparticles displaying multiple epitopes. If successful, this would enable us to co‐express the FepA‐encapsulin fusion proteins in *N. benthamiana* and eliminate the increased labor and time associated with producing the five fusion proteins individually. To assess this, we designed two additional encapsulin constructs, one containing an HA‐tag and the other a c‐Myc‐tag (E‐HA and E‐cMyc) flanked by flexible linkers (G_4_S) inserted at position K239‐D240, respectively (Fig. 6A). Even though both chloroplast targeting and cytosol targeting result in assembled nanoparticles, and L2 is displayed on the surface of chloroplast‐targeted nanoparticles, E‐HA and E‐cMyc were tested using only cytosolic targeting to ascertain that other peptides would be displayed on the surface of cytosolic nanoparticles as well. The individual expression of both proteins was assessed through transient expression in *N. benthamiana*, where both proteins accumulated to similar levels as E‐L2 (Fig. 6B). E‐L2, E‐cMyc, and E‐HA were only detected when probed with their respective antibodies, indicating that there is no non‐specific binding of the anti‐His, ‐c‐Myc, or ‐HA antibodies to encapsulin.

To assess whether the three different encapsulin monomers would co‐assemble into mosaic nanoparticles, E‐L2, E‐cMyc, and E‐HA were co‐expressed in *N. benthamiana*. Total soluble proteins were extracted and encapsulin nanoparticles were purified in two steps: IMAC to isolate proteins containing a His‐tag (E‐L2) followed by c‐Myc immunoprecipitation to isolate proteins that also contained a c‐Myc‐tag (E‐cMyc). As a negative control, tissue infiltrated with p19 alone underwent the same procedure.

Samples were separated by SDS/PAGE in triplicate and each replicate was immunoblotted with antibodies specific to either c‐Myc, HA, or L2. The encapsulin monomer (~31 kDa) was detected in all samples whether probed with anti‐c‐Myc, anti‐HA, or anti‐L2 antibodies, indicating that all three encapsulin monomers are co‐assembling in *N. benthamiana*. Higher molecular weight bands (~38–75 kDa) are visible in all three blots and likely correspond to assembled intermediates or adducts. No protein was detected in the p19‐negative control when probed with any of the antibodies (Fig. 6C).

To further confirm co‐assembly of the differentially tagged encapsulin monomers, the elutions from the c‐Myc immunoprecipitation were analyzed using immunogold labeling. Assembled nanoparticles were visualized surrounded by 10‐nm and 6‐nm gold particles, indicating the presence of c‐Myc and L2, or HA and L2, respectively, on the assembled nanoparticles, confirming that two differentially tagged encapsulin monomers can co‐assemble *in vivo*. For the negative controls where the primary antibodies were omitted, assembled nanoparticles were visualized; however, no gold particles were visible, suggesting that there is no nonspecific binding of the gold particles to the proteins (Fig. 6D). While it would be ideal to perform this assay in such a way that all three tags (c‐Myc, HA, and L2) could be detected simultaneously, this would require the addition of another gold particle and could potentially impede visualization of the assembled nanoparticles.

### E‐L2 is produced at high levels in transplastomic *Nicotiana tabacum*

**Fig. 7:**
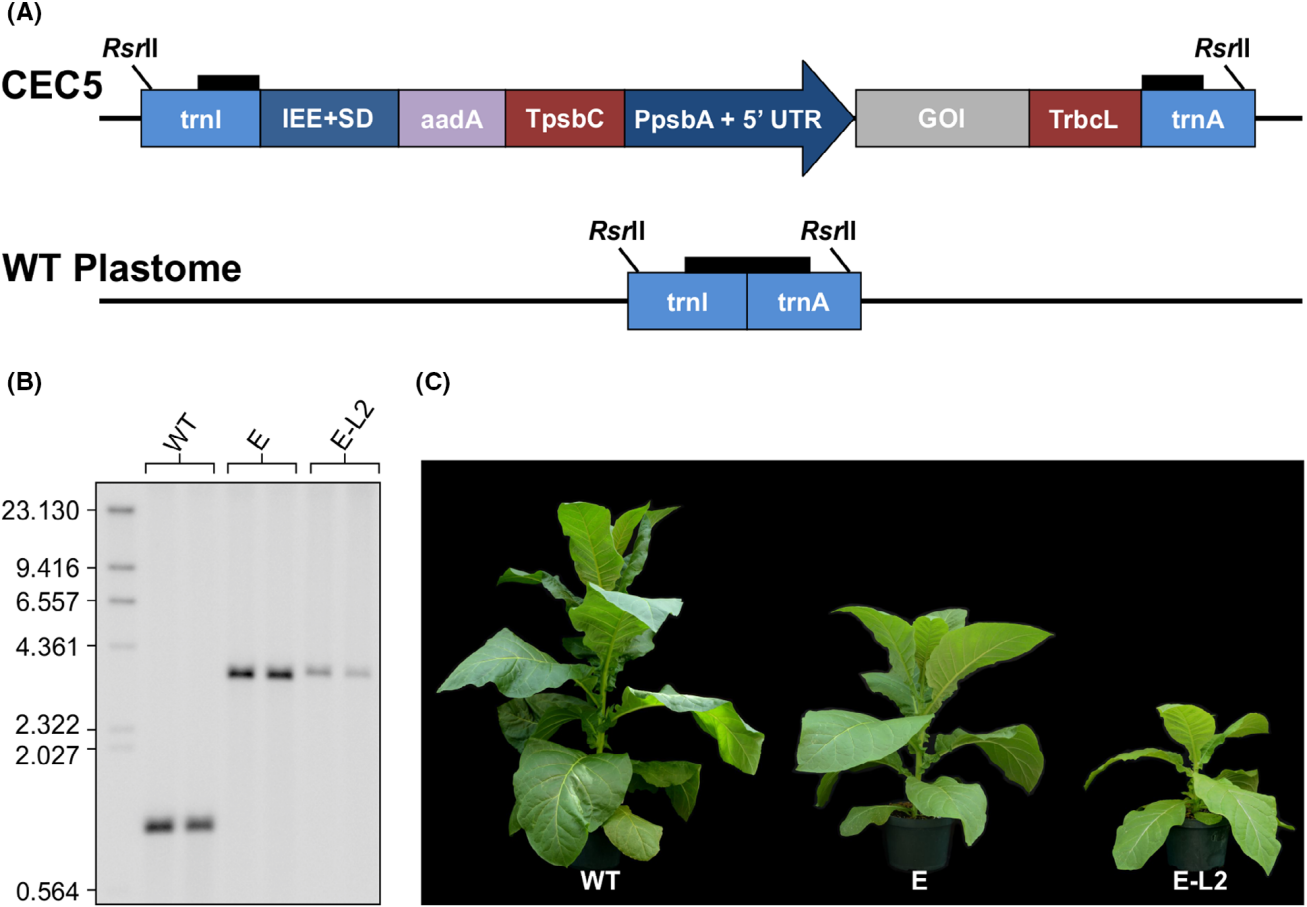
Generation of *encapsulin* and *E‐L2* transplastomic plants and confirmation of homoplasmy. (A) Chloroplast expression cassette 5 (CEC5) utilized for transplastomic expression of encapsulin and E‐L2 is depicted alongside the wild‐type *N. tabacum* plastome (WT plastome). The CEC5 vector contains various cis‐acting regulatory elements designed for integration into the transcriptionally active spacer region between the *trnI* and *trnA* genes of the *N. tabacum* plastome. Key components of the vector include the Intercistronic Expression Element fused to the Shine–Dalgarno sequence from the 5′ untranslated region (UTR) of bacteriophage T7 gene 10 (IEE + SD), the *aadA* gene for antibiotic resistance (aadA), the 3′ UTRs of psbC (TpsbC) and rbcL (TrbcL) from the white poplar plastome, and the promoter and 5′ UTR of the *N. tabacum psbA* gene (PpsbA +5′ UTR). The encapsulin and E‐L2 sequences were cloned into the Gene of Interest (GOI) region. Thick horizontal black lines indicate hybridization sites for probes used in Southern blot analysis. *Rsr*II restriction sites are indicated by thin black lines. (B) Southern blot analysis of two wild‐type (WT) plants, and two independent transplastomic lines for each of *encapsulin* (E) and *E‐L2* (E‐L2). Results are representative of two replicates of two individual lines. Chloroplast DNA was digested with *Rsr*II and separated by gel electrophoresis. The expected size for wild‐type *N. tabacum* (WT) is 1.054 kb, E is 3.319 kb, and E‐L2 is 3.423 kb. Numbers on the left hand side of the blot represent the size (kbp) of the DNA ladder. (C) T1 *encapsulin* (E) and *E‐L2* (E‐L2) transplastomic plants were grown synchronously with wild‐type (WT) *N. tabacum* and photographed at 8 weeks of age. Compared to the wild‐type control, expression of encapsulin and E‐L2 caused stunted growth in the transplastomic plants. Results are representative of five individual transplastomic lines. E, Encapsulin; FepA, Ferric enterobactin receptor; E‐L2, FepA loop 2–encapsulin fusion protein.

Following successful transient expression of encapsulin and FepA‐encapsulin fusion proteins in *N. benthamiana*, we decided to produce stably transformed *N. tabacum* plants expressing encapsulin and E‐L2. Agroinfiltration is time‐consuming and labor‐intensive, and utilizing *Agrobacterium* to facilitate recombinant protein expression poses safety concerns regarding the presence of endotoxins. To combat these issues, we sought to produce transplastomic *N. tabacum* lines where *encapsulin* and *E‐L2* are incorporated into the chloroplast genome. Transplastomic expression allows for high protein yield due to the high copy number of the transgene and the lack of gene silencing, and also enables transgene confinement due to maternal inheritance of the chloroplast genome [43, 44]. *Encapsulin* and *E‐L2* were cloned into one of our in‐house chloroplast expression cassettes (Fig. 7A) and were incorporated into the chloroplast genome between *trnI* and *trnA* using biolistic transformation [45]. Successful transformants were selected following three rounds of regeneration on selective media and integration of the transgene was confirmed via Southern blot analysis (Fig. 7B). Compared to wild‐type *N. tabacum*, *encapsulin* and *E‐L2* transplastomic plants displayed slightly stunted growth (Fig. 7C).

**Fig. 8:**
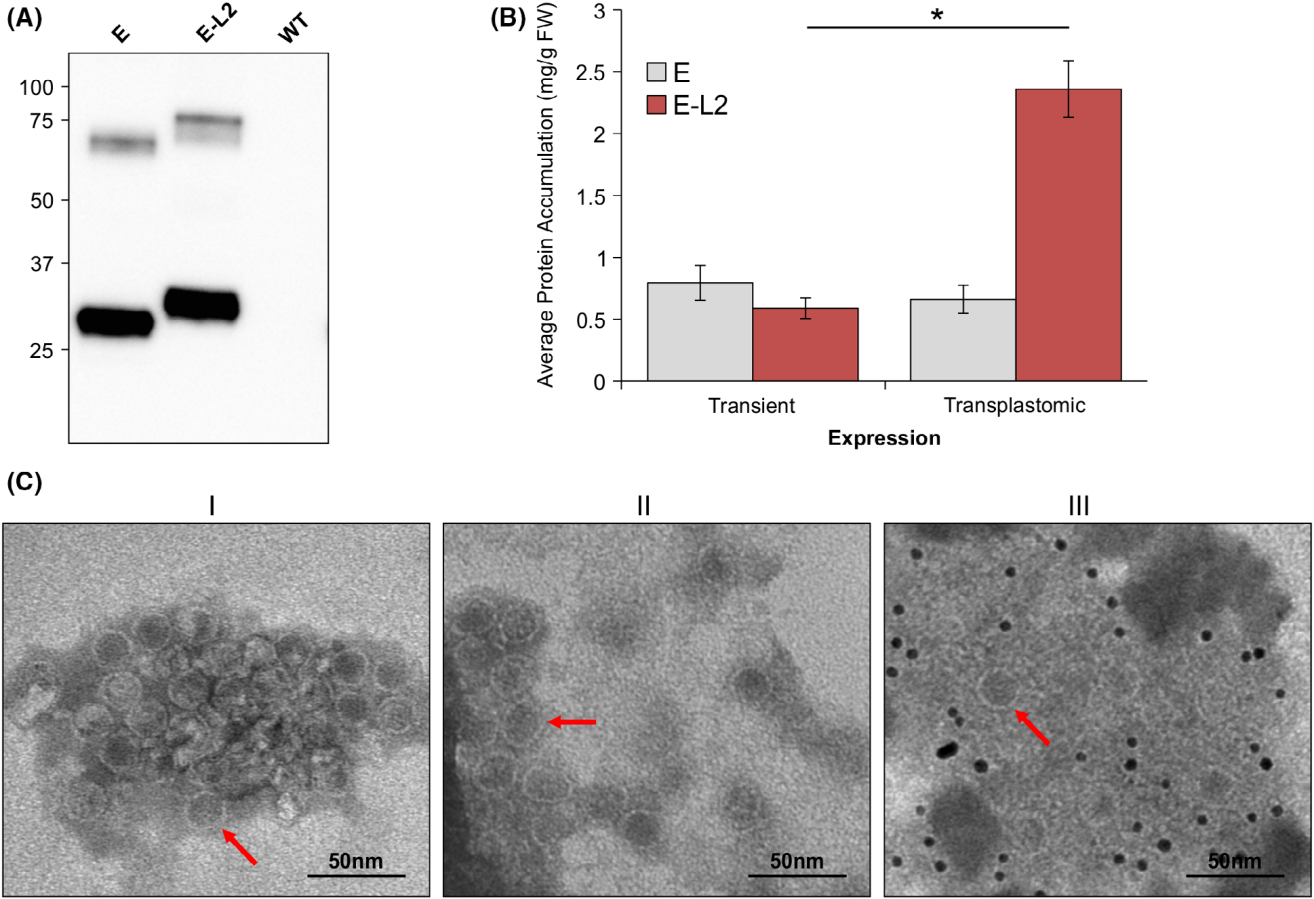
E and E‐L2 accumulate and assemble in transplastomic *N. tabacum*. (A) Accumulation of E and E‐L2 in transplastomic *N. tabacum*. Results are representative of three replicates. Lanes were loaded with total soluble protein extracted from 0.05 mg of leaf tissue. Proteins were detected using an anti‐His primary antibody (1:5000). Numbers on the left hand side of the blot represent the size (kDa) of the molecular weight markers. The expected molecular weight (kDa) of E is 31.7 and E‐L2 is 35.5. (B) Average recombinant protein accumulation levels in mg·g^−1^ leaf tissue fresh weight (mg·g^−1^ FW) in transplastomic plants expressing E and E‐L2. The transiently expressed proteins were targeted to the chloroplast and samples were collected on Day 3 postinfiltration. *N* = 15. For the transplastomic samples, *N* = 8 and *N* = 6 for E and E‐L2, respectively. Accumulation levels were averaged across biological replicates (± standard error of the mean) and significance (*P‐value <0.05) was determined by a one‐tailed Student's *t*‐test for unequal variance. (C) Purified proteins were negatively stained and examined using a JEOL JEM‐1400Flash (JEOL Ltd.) microscope operated at 80 kV. All images were taken at 80 000× magnification. Size‐bars represent 50 nm. Red arrows indicate assembled nanoparticles. I – E. II – E‐L2. III – E‐L2 immunolabeled with an anti‐L2 primary antibody. Gold particles (6 nm) surround nanoparticles indicating that the L2 epitope is displayed on the nanoparticle surface. Images are representative of the entire grid and similar results were visualized across two replicates. E, Encapsulin; FepA, Ferric enterobactin receptor; E‐L2, FepA loop 2–encapsulin fusion protein; L2, FepA loop 2; WT, Wild‐type *N. tabacum*.

Unlike with chloroplast‐targeted transient expression in *N. benthamiana*, transplastomic expression of encapsulin and E‐L2 in *N. tabacum* resulted in the presence of a single band, providing further support that the double‐banding pattern observed upon transient expression is due to retention of the chloroplast transit‐peptide (Fig. 8A). In *N. tabacum*, both encapsulin and E‐L2 accumulated to high levels (0.66 and 2.36 mg·g^−1^ FW), and in the case of E‐L2, there was almost a fivefold increase in protein accumulation compared to transient expression (0.59 mg·g^−1^ FW) (Fig. 8B). As seen in *N. benthamiana*, both proteins assembled into nanoparticles and using immunogold labeling, the L2 epitope was confirmed to be displayed on the exterior surface of encapsulin (Fig. 8C), indicating that both expression platforms are suitable to produce properly‐assembled encapsulin nanoparticles.

## Discussion

In the pursuit to develop effective subunit vaccines against *Salmonella*, researchers have primarily focused on surface‐exposed proteins that are essential for *Salmonella's* survival and virulence, such as type 1 fimbriae and proteins from the Type III secretion system [46, 47]. While vaccines based on these proteins provided some degree of protection, they failed to eliminate *Salmonella* from the internal organs. In an effort to develop a more effective subunit vaccine against *Salmonella*, this study focused on the surface‐exposed protein FepA, which is involved in *Salmonella* iron acquisition. Previous studies have highlighted the importance of *Salmonella* iron acquisition proteins and their potential for vaccine candidacy [13, 15, 48, 49, 50].

In recent years, protein nanoparticles have gained increasing attention as antigen display platforms for vaccine development [25, 51, 52, 53]. Due to their symmetric and repetitive pathogen‐like structure, they can display antigens in a highly organized manner and overcome the low immunogenicity typically associated with monomeric antigens used in recombinant vaccines [18, 19, 54]. Their large supramolecular structure can also confer adjuvant‐like properties, which can lead to the induction of a robust immune response [55], and they can efficiently present epitopes to the mucosal immune system, which is of particular importance for bacterial pathogens, such as *Salmonella*, which colonize the mucosa [31]. Therefore, displaying *Salmonella* epitopes on protein nanoparticles, such as encapsulin, could be a promising approach for developing an effective vaccine.

Of the six modification sites within the encapsulin monomer, position 138–139 has been the most extensively utilized for vaccine design and drug delivery [22, 23, 24, 25]. While several studies have reported successful genetic modification of position 138–139, many have not indicated the identity of the amino acids at this position, nor do they indicate the encapsulin sequence used. This poses difficulties as two different reference sequences exist for encapsulin from *T. maritima*: One reported in Sutter *et al*. [21], and one published on Protein Data Bank (PDB: 3DKT) by Sutter *et al*., the former having three additional N‐terminal amino acids. Most studies reference Sutter *et al*. [21] when discussing the modification sites they utilized; however, since there are discrepancies in the amino acid sequences published by this group, this poses a challenge when determining which amino acids position 138–139 correspond to, as this would vary depending on the sequence used. The exception is a study by Michel‐Souzy *et al*. [22] where they indicated the amino acid identity of the modification sites used. However, they used the sequence from Sutter *et al*. [21], whereas we used the PDB sequence; therefore, their position E138‐E139 does not correspond to the same amino acids as our position K138‐I139. Since it is unclear which sequence was used in other reports, it is uncertain whether the modification site K138‐I139 we used is novel. Nevertheless, the insertion position K239‐D240 was not reported previously.

This current work is the first time encapsulin has been produced in plants. We have shown that plant‐produced encapsulin accumulates to high levels and no significant change in accumulation was observed upon the fusion of most FepA epitopes. In many cases, adding sequence to a protein could affect protein assembly, stability, and solubility [56, 57, 58]; however, this was not observed for any of the chloroplast‐targeted fusion proteins. Similarly, cytosol‐targeted E‐L2, E‐HA, and E‐cMyc all accumulated to similar levels, suggesting that the addition of HA‐ and c‐Myc‐tags to encapsulin does not affect its stability. However, for the cytosol‐targeted fusion proteins, E‐L8 had a significantly lower accumulation level, and E‐L10 could not be expressed, suggesting that these epitopes may impact the stability of encapsulin targeted to the cytosol. *In planta* production is an attractive platform for vaccine production, particularly livestock vaccines, as plants are easy to grow, highly scalable, inexpensive to maintain, and have the potential for oral delivery through the addition to animal feed [31, 59, 60]. However, one problem commonly encountered with plant production of recombinant proteins is low yield. An essential goal for the development of livestock vaccines is to keep production costs as low as possible, as farmers are unlikely to invest in expensive preventative medications [61]. In this study, we found high accumulation of FepA‐encapsulin fusion proteins, with the accumulation of transiently expressed E‐L4 and transplastomically expressed E‐L2 at 0.7 mg·g^−1^ and 2.4 mg·g^−1^ FW, respectively.

Two distinct proteins were present in transiently expressed chloroplast‐targeted encapsulin and FepA‐encapsulin samples. Analysis of these proteins using LC–MS confirmed that the two proteins correspond to the mature protein and the immature protein with the chloroplast transit peptide still intact. Nuclear expressed proteins that reside in the chloroplast are typically delivered to the chloroplast surface by cytosolic chaperones that recognize their transit peptide and maintain the protein in an unfolded conformation suitable for import. The proteins then enter the chloroplast via translocon complexes in the outer and inner envelope membranes. These complexes recognize the transit peptide and transport the protein into the chloroplast stroma where the transit peptide is removed via a stromal processing peptidase (SPP) [62]. It is possible that the incomplete cleavage of the transit peptide we observed with transient expression is due to nanoparticle assembly in the cytosol, as assembled intermediates would be in a folded conformation and thus would not be suitable for import into the stroma. It could also be the result of an abundance of recombinant protein in the chloroplast such that the SPP is unable to cleave the transit peptides efficiently. Regardless of the reason for its retention, our results indicate that the presence of the transit peptide does not impact encapsulin assembly, as we observed assembled nanoparticles for encapsulin and all FepA‐encapsulin fusion proteins. Moreover, the transit peptide does not appear to interfere with epitope display on encapsulin, as the L2 peptide was found to be displayed on the exterior surface of assembled encapsulin. The chloroplast transit peptide is located on the N terminus of encapsulin, which folds into the interior of the assembled nanoparticle [21], which explains why its presence may not impede epitope display, and is therefore unlikely to interfere with the epitopes' interaction with the immune system.

Previous studies have shown *in vitro* assembly of mosaic nanoparticles displaying two or more antigens [63, 64, 65]. However, this had not been demonstrated with encapsulin nanoparticles. Here, we have shown that differentially tagged encapsulin monomers co‐assemble *in vivo* to form mosaic nanoparticles. This finding shows promise for the production of a nanoparticle carrying multiple epitopes. Rather than producing the FepA‐encapsulin fusion proteins individually, different constructs could be co‐expressed such that the resulting assembled nanoparticles may contain all or a majority of the target epitopes. One drawback to this approach is that the nanoparticles will assemble randomly; thus, there would be no control over the quantity of the different epitopes as they may be incorporated into nanoparticles to varying degrees, with assembly potentially favoring some proteins. However, even though the population of assembled nanoparticles is heterogenous, the protein sample would still contain all the epitopes that were co‐expressed.

While previous studies have primarily utilized *E. coli* for encapsulin expression, we evaluated its expression in plants for a potential use as an oral vaccine against *Salmonella*. Here, we have used *Nicotiana* spp., which are widely explored systems for the production of recombinant proteins. However, due to the presence of toxic secondary metabolites such as alkaloids, including nicotine, they are not currently considered edible plants which thereby limits their use for the development of oral vaccines [66]. While edible plants, such as lettuce, have been investigated as delivery vehicles for oral vaccines, *N. tabacum* has advantages such as ease of transformation and large biomass allowing for high protein yield per plant [67]. To mitigate toxicity issues due to high alkaloid content, we used a low‐alkaloid *N. tabacum* cultivar 81 V9 [66], making it more suitable for oral administration of plant tissue. In fact, mice that consumed 81 V9 low‐alkaloid tobacco at concentrations up to 30% of their diet exhibited no observed toxicity and gained as much weight as their control counterparts [68]. Therefore, although there are regulatory hurdles that remain, there is potential to produce edible vaccines using low‐alkaloid *N. tabacum* lines.

For veterinary vaccines, edible vaccines would provide several benefits over injection. First would be the technical ease of vaccine production and administration. There could potentially be no need for protein extraction and purification; rather, the plant tissue could simply be lyophilized and mixed into the livestock feed at designated immunization intervals. This would greatly reduce production costs and eliminate the need for laborious and expensive administration by a veterinarian [8, 30]. Administration of the vaccine at designated time intervals could also reduce the risk of oral tolerance to the vaccine [69]. While this would allow for more efficient vaccination, it would be difficult to manage the dose that each bird receives as they would not all ingest the same quantity. This is less problematic for animals as a wide dosage range is generally acceptable for livestock vaccines, unlike human vaccines where dosage is strictly regulated [31]. For example, an oral papaya vaccine against *Taenia solium* induced a strong immune response in pigs even when administered across a two‐log dose range (1, 10, or 100 μg) [70]. This flexibility underscores the potential of edible vaccines for animals, as effective immunization can often be achieved within a wide range of doses. Moving forward, determining broad minimum and maximum effective doses will be essential for optimizing this approach.

A second benefit to oral administration is the direct delivery of the vaccine to the mucosal tissues which would increase secretory IgA titers in the mucosa [71]. This is critical for pathogens, such as *Salmonella*, that enter the host via mucosal tissues, as secretory IgA helps prevent them from attaching to the mucous layer [72, 73]. However, successful oral vaccines must survive the low pH and high enzymatic activity within the gastrointestinal tract, such that the antigen is transported across the mucosal barrier where it can activate antigen‐presenting cells [74]. Thus, formulation steps may be required to protect lyophilized leaf tissue in low gastric pH and ensure that intact proteins are delivered to the intestinal mucosa.

While this study highlights the potential of encapsulin as a display platform for the development of a candidate *Salmonella* vaccine, further work needs to be done to characterize the immune response induced by FepA‐encapsulin fusion proteins and assess whether the resulting antibodies can prevent *Salmonella* colonization. Recent studies have investigated the production of oral *Salmonella* vaccine candidates using outer membrane proteins [10]. Ijaz *et al*. (2025) expressed the *Salmonella* outer membrane protein C (OmpC) antigen in *N. tabacum* and found that mice immunized orally and subcutaneously with total soluble protein or purified OmpC protein generated a high OmpC‐specific immune response and elicited a significant protective effect in mice upon challenge with *Salmonella* [10].

Moving forward, an immunization and *Salmonella* challenge experiment with the FepA‐encapsulin proteins will be performed in chickens to characterize the immune response induced by FepA‐encapsulin fusion proteins and assess whether the resulting antibodies are able to prevent *Salmonella* colonization. It is possible that a vaccine targeting a single FepA epitope may not be sufficient to produce a protective immune response; therefore, a vaccine containing a mixture of FepA loops will be investigated, utilizing our strategy of co‐expression to produce a mosaic multivalent vaccine. While there is further work to be done to investigate the efficacy of FepA‐encapsulin fusion proteins in protecting against *Salmonella*, this study provides a promising foundation for the use of plant‐produced encapsulin for epitope display, and the potential for a plant‐produced multivalent *Salmonella* vaccine that could overcome the cost, efficacy, and administration limitations associated with those currently available.

## Materials and methods

### Construct design and cloning

The encapsulin sequence was based on the encapsulin monomer from *T. maritima* published on Protein Data Bank (PDB: 3DKT), with the removal of the N‐terminal methionine. *Salmonella enterica* serovar Enteritidis (GenBank accession no. AM933172.1) was used for the identification of the FepA antigenic peptides. Structural analysis of encapsulin and FepA was performed using PyMOL (The PyMOL Molecular Graphics System) and Phyre2 [75].

For transient expression, the encapsulin and FepA‐encapsulin fusion gene constructs were codon‐optimized for nuclear expression in *N. benthamiana* and were synthesized by Bio Basic Inc. (Markham, ON, Canada). All constructs included flanking *Bsa*I sites to allow for Golden Gate cloning into plant expression vectors. The genes were cloned into two modified pCamLiteGoldenGate‐X (pCLGG‐X) plant expression vectors [76], enabling the resulting proteins to be targeted to the chloroplast and the cytosol using Golden Gate cloning [77]. Both vectors contain a double‐enhanced *35S* promoter [78] and tobacco cryptic upstream promoter translational enhancer (*tCUP*) [79] for high expression, and the *Nos* terminator [80]. The chloroplast vector contains the chloroplast transit peptide from the small subunit of RuBisCo (RBC‐S‐TP), to target proteins to the chloroplast stroma [81]. Both vectors also contain two glycine residues to stabilize the N terminus of the mature protein, and the cytosol vector contains a methionine at the N terminus as well (MGG). The cloned vectors were transformed into NEB 10‐beta competent *E. coli* cells for plasmid propagation, and transformation was confirmed by selection and colony PCR. All plasmids were subsequently isolated and transformed into *Agrobacterium tumefaciens* EHA‐105 cells for transient expression in *N. benthamiana* [82].

### Growth and care of *N. benthamiana* plants

Wild‐type plants were grown in a walk‐in growth chamber at 22 °C with 65% relative humidity under a 16‐h photoperiod at a light density of ~100 μmol m^−2^ s^−1^. Plants were grown in 4‐inch pots with PRO‐MIX BX soil and were watered with 20 : 8 : 20 fertilizer (N : P : K) at a concentration of 0.25 g·L^−1^ of water.

### Expression of recombinant proteins in *N. benthamiana*

Agroinfiltration of *N. benthamiana* leaves was performed as previously described [83]. Briefly, *Agrobacterium* cultures containing the genes of interest were mixed 1:1 with cultures containing *p19*, a suppressor of post‐transcriptional gene silencing from the *Cymbidium* ringspot virus [84], and co‐infiltrated into the abaxial side of 6‐ to 8‐week‐old *N. benthamiana* leaves using a needless syringe. *Agrobacterium* containing *p19* alone was used as a negative control.

For the time course to assess recombinant protein accumulation, three leaves on five *N. benthamiana* were agroinfiltrated. One leaf disk was collected from each infiltrated area on Days 3, 5, and 7 postinfiltration and pooled across all five plants on each collection day. The tubes containing the tissue were then flash‐frozen in liquid nitrogen and stored at −80 °C.

Experiments using five plants as biological replicates were repeated two (time course) or three times (protein quantification) to allow for 10 or 15 biological replicates, respectively.

### Analysis of recombinant proteins

Total soluble proteins were isolated from infiltrated leaf tissue as described previously [83]. Briefly, frozen leaf disks were homogenized using a TissueLyser (QIAGEN, Venlo, Netherlands), and total soluble proteins were extracted using plant protein extraction buffer containing 1× phosphate‐buffered saline (PBS), 0.1% (v/v) Tween‐20, 2% (w/v) polyvinylpolypyrrolidone (PVPP), 100 mm ascorbic acid, 1 mm ethylenediaminetetraccetic acid (EDTA), 1 mm of phenylmethanesulfonylfluoride (PMSF), and 1 μg·mL^−1^ leupeptin [83]. The proteins were separated by SDS/PAGE and transferred to polyvinylidene difluoride (PVDF) membranes. The recombinant proteins were detected using mouse anti‐His monoclonal primary antibody (Takara Bio USA Inc., San Jose, CA, USA, product number: 631212) and horseradish peroxidase‐conjugated goat anti‐mouse IgG secondary antibody (Bio‐Rad, Hercules, CA, USA, product number: 1706516). The recombinant proteins were visualized using the Clarity Western ECL detection kit (Bio‐Rad, Hercules, CA, USA, product number: 1705061) and the MicroChemi 4.2 (DNR Bio‐Imaging Systems Ltd., Marienbongard, AC, Germany) and quantified by densitometry using GelQuant software (DNR Bio‐Imaging Systems Ltd., Marienbongard, AC, Germany). The quantification was performed within a linear detection range where the intensity of both chloroplast bands, or the single cytosol band, were compared to a dilution series consisting of known amounts of purified encapsulin, which was previously quantified against Bovine Serum Albumin (BSA) on a gel stained with GelCode Blue Reagent (Thermo Fisher Scientific, Waltham, MA, USA). The same encapsulin standard was used for all blots to account for variability. Accumulation levels in mg·g^−1^ of leaf fresh weight (FW) were calculated based on leaf mass, volume of plant protein extraction buffer, dilution factor of protein extract, and volume of extract loaded on the gel. Accumulation levels were averaged across biological replicates and significance was determined by ANOVA followed by a Tukey's *post hoc* test.

### Purification of recombinant proteins

The recombinant proteins were first purified by Immobilized Metal Affinity chromatography (IMAC) using Nickel Sepharose 6 Fast Flow resin (GE Healthcare, Chicago, IL, USA). Infiltrated *N. benthamiana* leaves were flash‐frozen, and soluble proteins were extracted in 5–10 volumes (v/w) of plant protein extraction buffer, with 20–40 mm imidazole, per gram of leaf tissue. The homogenate was clarified twice by centrifugation at 20 000 ***g*** at 4 °C for 15 min. Resin (0.5 mL) was packed into a 9 mL Poly‐Prep Chromatography column (Bio‐Rad, Hercules, CA, USA), washed, and equilibrated with buffer (1× PBS, 20–40 mm imidazole) following the manufacturers' protocol. The clarified extract was then added to the column and passed through via gravity flow. Once the extract had passed through, the column was washed twice with 2.5 mL of wash buffer (1× PBS, 20–40 mm imidazole). The bound recombinant protein was then eluted by adding 2.5 mL of elution buffer (1× PBS, 500 mm imidazole). Eluate was collected in 0.5 mL fractions. The eluted fractions were analyzed using SDS/PAGE followed by staining with GelCode Blue reagent (Thermo Fisher Scientific, Waltham, MA, USA).

The fractions containing the most recombinant protein were then further purified by size‐exclusion chromatography (SEC) using a Superose 6 Increase 10/300 GL column (GE Healthcare, Chicago, IL, USA) to separate the assembled nanoparticles from unassembled recombinant proteins and any remaining endogenous plant proteins.

### Transmission electron microscopy and immunogold labeling

To examine nanoparticle assembly, 5 μL of purified recombinant protein was spotted onto formvar/carbon coated 400 mesh copper grids (Electron Microscopy Sciences, Hatfield, PA, USA, product number: FCF400‐Cu‐50) and negatively stained with 2% (w/v) uranyl acetate (UA). The grids were imaged using a JEOL JEM‐1400Flash microscope (JEOL Canada, Ltd., Saint‐Hubert, QC, Canada).

To assess whether FepA epitopes are displayed on the exterior surface of assembled encapsulin, a custom primary antibody against FepA loop 2 (L2) was produced by Bio Basic Inc. (Markham, ON, Canada). Briefly, two New Zealand rabbits were immunized with four doses (400 μg·dose^−1^) of synthesized L2 peptide on Days 1, 15, 29, and 43. A test bleed was collected on Day 36, and the final bleed was collected on Day 50. The antibody titers of both bleeds were assessed via ELISA. Antibodies were purified from the final bleed sera using a protein affinity resin in which the L2 peptide was coupled to CNBR‐activated Sepharose.

Immunogold labeling was performed on purified E‐L2 nanoparticles. First, formvar/carbon coated 400 mesh copper grids were spotted with purified E‐L2 protein and blocked with 0.3% BSA for 15 min. The grids were incubated with a 1/650 dilution of rabbit anti‐L2 primary antibody for 1 h. After five washes with 0.03% BSA, the grids were incubated for 1 h with a 1/15 dilution of 6‐nm colloidal gold‐conjugated goat anti‐rabbit secondary antibody (Electron Microscopy Sciences, Hatfield, PA, USA, product number: 170–6515). The grids were then washed five times with 0.03% BSA and three times with distilled water. Finally, the grids were negatively stained with 2% UA and examined using a JEM‐1400 microscope (JEOL Canada, Ltd., Saint‐Hubert, QC, Canada).

### LC–MS identification of cytosol‐ and chloroplast‐targeted E‐L2

IMAC‐purified cytosol‐ and chloroplast‐targeted E‐L2 were concentrated to a volume of 100 μL using a Vivaspin™ 500 30 K MWCO concentrator (Cytiva, Marlborough, MA, USA). The concentrated samples were washed with 1 mL of 10 mm ammonium acetate and concentrated again to a volume of 100 μL. This was repeated using 2.5 mm ammonium acetate. The concentrated samples were removed and diluted to 1 mg·mL^−1^ with 2.5 mm ammonium acetate.

LC–MS analysis was conducted as described previously [85]. Intact protein analysis was performed under denaturing conditions on a Thermo Q‐Exactive Orbitrap mass spectrometer coupled to an Agilent 1290 HPLC system. Sample (5 μL) was injected onto a Zorbax 300 SB‐C8 RRHD column maintained at 80 °C (2.1 × 100 mm, 1.8 μm; Agilent, Santa Clara, CA, USA). Mobile phase A (0.1% formic acid in LC–MS grade H_2_O, Thermo Fisher Scientific, Waltham, MA, USA) started at 0% and was held for 1 min. Mobile phase B (0.1% formic acid in LC–MS grade acetonitrile, Thermo Fisher Scientific, Waltham, MA, USA) was then increased to 100% over 9.5 min, maintained for 0.5 min, and returned to 0% over 1 min. Samples were analyzed using a 70 000 resolution full MS analysis, in the mass range of m/z 650–4000 with 5 × 10^5^ AGC and 512 max IT. The following conditions were used for heated electrospray ionization (HESI): capillary voltage, 3.75 kV; capillary temperature, 400 °C; sheath gas, 25 arbitrary units; auxiliary gas, 8 units; probe heater temperature, 400 °C; and S‐Lens RF level, 90. The data was analyzed using the Thermo Xcalibur software. The intact mass was calculated as the average of each charge state as

$$\text{Intact mass}=\frac{1}{n}\sum_{i=1}^{n}{\left(m/z\right)}_{i}\cdot z_{i}-z_{i}\cdot H^{+}$$

where *z* represents the charge, *m/z* represents the experimental mass to charge ratio and H^+^ represents the mass of a protein (1.00726 Da).

### Co‐expression and assembly of differentially tagged encapsulin monomers

To assess the co‐assembly of differentially tagged encapsulin monomers in *N. benthamiana*, two encapsulin constructs were designed with either an HA‐tag or a c‐Myc‐tag (E‐HA and E‐cMyc) flanked by flexible linkers (G_4_S) inserted at position K239‐D240, respectively. Both constructs were cloned into pCLGG‐X plant expression vectors designed to target the proteins to the cytosol. The accumulation of E‐HA and E‐cMyc was assessed using the methods described above. E‐HA, E‐cMyc, and E‐L2 crude extracts were run in triplicate on an SDS/PAGE alongside an in‐house protein standard which contains an HA‐, c‐Myc‐, and His‐tag. Following transfer to a PVDF membrane, the blot was cut in three and each third was probed with a mouse anti‐hemagglutinin (HA) primary antibody (Sigma‐Aldrich, Oakville, ON, Canada, product number: H3663), mouse anti‐c‐Myc primary antibody (Genscript, Piscataway, NJ, USA, product number: A00864), or mouse anti‐His monoclonal primary antibody (Takara Bio USA Inc., San Jose, CA, USA, product number: 631212). Following confirmation of expression of E‐HA and E‐cMyc, *Agrobacterium* containing E‐HA, E‐cMyc, and E‐L2 were co‐infiltrated in *N. benthamiana*, using the method described above, in a 1:1:1:1 ratio with *Agrobacterium* containing p19. Leaves were collected from co‐infiltrated plants, flash‐frozen, and total soluble proteins were extracted following the method described above.

Following extraction, the recombinant proteins were purified using a two‐step purification method. First, they were purified using IMAC as described above, and the eluted proteins underwent three rounds of dialysis in 1 L 1× PBS. The dialyzed proteins were then concentrated to ~0.5 mL using a Vivaspin™ 20 10 K MWCO concentrator (Cytiva, Marlborough, MA, USA). The proteins were then further purified using an Anti‐c‐Myc Immunoprecipitation Kit (Sigma‐Aldrich, Oakville, ON, Canada, product number: IP0020) following the manufacturers protocol. Samples from each of the purification steps, as well as crude extract, were analyzed via western blot using a mouse anti‐HA primary antibody (Sigma‐Aldrich, Oakville, ON, Canada, product number: H3663), mouse anti‐c‐Myc primary antibody (Genscript, Piscataway, NJ, USA, product number: A00864), and rabbit anti‐L2 primary antibody (Bio Basic Inc., Markham, ON, Canada) along with either a horseradish peroxidase‐conjugated goat anti‐mouse IgG secondary antibody (Bio‐Rad, Hercules, CA, USA, product number: 1706516) or goat anti‐rabbit IgG secondary antibody (Bio‐Rad, Hercules, CA, USA, product number: 1706515). All the procedures describe above were also performed with *N. benthamiana* leaves infiltrated with p19 alone to serve as a negative control.

The elutions from the anti‐c‐Myc immunoprecipitation of the co‐infiltration were also analyzed via immunogold labeling following the procedure described above. Samples were incubated with a mix of anti‐c‐Myc and anti‐L2 primary antibodies, or anti‐HA and anti‐L2 primary antibodies, followed by a mix of 10‐nm colloidal gold‐conjugated goat anti‐mouse (Electron Microscopy Sciences, Hatfield, PA, USA, product number: 25129) and 6‐nm colloidal gold‐conjugated goat anti‐rabbit (Electron Microscopy Sciences, Hatfield, PA, USA, product number: 170–6515) secondary antibodies.

### Generation of transplastomic tobacco plants

For transplastomic expression in *N. tabacum*, the *encapsulin* and *E‐L2* genes were codon‐optimized for expression in *E. coli*, as the chloroplast is bacterial in origin. Both genes were synthesized by Twist Bioscience (South San Francisco, CA, USA) and included *Nhe*I and *Not*I sites at the N‐ and C termini, respectively, to allow for cloning into our in‐house CEC5 expression vector [45]. The CEC5 vector was designed to integrate the gene of interest into the transcriptionally active spacer region between the *trnI* and *trnA* genes of the tobacco plastome. The vector contains cis‐acting elements including: The intercistronic expression element with the Shine‐Dalgarno sequence from the 5′ UTR of bacteriophage T7 gene 10 fused to the 3′ end (IEE + SD), to promote efficient mRNA processing and protein expression [86]; the aminoglycoside adenylyltransferase (*aadA*) gene, which confers resistance to streptomycin/spectinomycin [87, 88] for use as a selectable marker; the 3′ UTR regions of *rbcL* (Tr*bcL*) and *psbC* (T*psbC*) plastid genes from the White Poplar plastome [89, 90]; and the promoter for the PSII protein D1 (P*psbA*) fused to the 5′ UTR of the tobacco plastid *psbA* gene [91, 92]. The *encapsulin* and *E‐L2* sequences were digested with *Nhe*I and *Not*I and introduced into the pCEC5 vector through direct cloning into the corresponding restriction sites [72].

Transplastomic *N. tabacum* plants (cv. 81 V9) were generated via the biolistic method [92]. After three rounds of regeneration on selective media containing 500 μg·mL^−1^ spectinomycin, positive transformants were confirmed via PCR and transferred to rooting media. Upon root formation, the plantlets were transplanted to soil. Integration of the transgene into the *N. tabacum* plastome was confirmed by Southern blot analysis, as described previously [43]. Briefly, DNA from transplastomic plants, along with wild‐type *N. tabacum*, was digested with the *Rsr*II restriction enzyme and separated on a 0.8% agarose gel. A DIG‐labeled probe was amplified with primers Probe‐F (5′‐caccacggctcctctcttctcg‐3′) and Probe‐R (5′‐ttcctacggggtggagatgatgg‐3′), which target the intergenic space region between the *trnI* and *trnA* genes in the tobacco plastome. Recombinant protein expression was confirmed through immunoblotting, as described above, using a mouse anti‐His monoclonal primary antibody (Takara Bio USA Inc., San Jose, CA, USA, product number: 631212). Recombinant protein accumulation levels were quantified as described above, and significance was determined by a one‐tailed Student's *t*‐test for unequal variance.

## Conflict of interest

The authors declare no conflicts of interest.

## Author contributions

The manuscript was written through contributions of all authors. All authors have given approval to the final version of the manuscript. CAC, AK, CPG, and SS conducted protein structural analyses and designed constructs. CAC conducted all expression, purification and assembly experiments and wrote the manuscript draft. JBR conducted mass spectrometry, MSD and RM conceived the project, obtained funding and edited the manuscript.

## Data Availability

All data supporting the findings of this study are freely available upon request.
